# The gastrointestinal microbiota in the development of ME/CFS: a critical view and potential perspectives

**DOI:** 10.3389/fimmu.2024.1352744

**Published:** 2024-03-28

**Authors:** Andreas Stallmach, Stefanie Quickert, Christian Puta, Philipp A. Reuken

**Affiliations:** ^1^ Department of Internal Medicine IV (Gastroenterology, Hepatology, and Infectious Diseases), Jena University Hospital, Jena, Germany; ^2^ Department of Sports Medicine and Health Promotion, Friedrich-Schiller-University Jena, Jena, Germany; ^3^ Center for Sepsis Control and Care (CSCC), Jena University Hospital/Friedrich-Schiller-University Jena, Jena, Germany; ^4^ Center for Interdisciplinary Prevention of Diseases Related to Professional Activities, Jena, Germany

**Keywords:** ME/CFS, FMT, post-COVID, gastrointestinal microbiome, fatigue

## Abstract

Like other infections, a SARS-CoV-2 infection can also trigger Post-Acute Infection Syndromes (PAIS), which often progress into myalgic encephalomyelitis/chronic fatigue syndrome (ME/CFS). ME/CFS, characterized by post-exercise malaise (PEM), is a severe multisystemic disease for which specific diagnostic markers or therapeutic concepts have not been established. Despite numerous indications of post-infectious neurological, immunological, endocrinal, and metabolic deviations, the exact causes and pathophysiology remain unclear. To date, there is a paucity of data, that changes in the composition and function of the gastrointestinal microbiota have emerged as a potential influencing variable associated with immunological and inflammatory pathways, shifts in ME/CFS. It is postulated that this dysbiosis may lead to intestinal barrier dysfunction, translocation of microbial components with increased oxidative stress, and the development or progression of ME/CFS. In this review, we detailed discuss the findings regarding alterations in the gastrointestinal microbiota and its microbial mediators in ME/CFS. When viewed critically, there is currently no evidence indicating causality between changes in the microbiota and the development of ME/CFS. Most studies describe associations within poorly defined patient populations, often combining various clinical presentations, such as irritable bowel syndrome and fatigue associated with ME/CFS. Nevertheless, drawing on analogies with other gastrointestinal diseases, there is potential to develop strategies aimed at modulating the gut microbiota and/or its metabolites as potential treatments for ME/CFS and other PAIS. These strategies should be further investigated in clinical trials.

## Introduction

Chronic fatigue syndrome/myalgic encephalomyelitis (CFS/ME) is a complex and disabling illness with an unclear etiology and pathogenesis and is characterized by persistent and disabling fatigue, exercise intolerance, post-exertional malaise, cognitive difficulties, and musculoskeletal/joint pain (1). Various triggers for ME-CFS are known, with infections being the most common occurrence (2). Infection with the SARS-CoV-2 virus is currently understood to be the most prevalent cause of this clinical picture. Consequently, the global COVID-19 pandemic has led to a significant increase in ME/CFS patients, affecting millions of individuals, including children and young adults (3, 4). A global prevalence of ME/CFS of approximately 0.8–3.3% was described in a meta-analysis by Johnston and Brenu; these estimates are based on clinical studies and self-assessments from those affected (5). However, studies by the National Institute for Health and Care Excellence (NICE) indicating a prevalence of 0.2-0.4% of the world’s population suffering from ME/CFS appear to be more reliable (6) The question remains whether the percentage is 0.2% or 3.3%. Given the lack of proven diagnostic markers and specific therapy for ME/CFS, coupled with treatment costs for ME/CFS patients being 50% higher than those for patients with conditions like multiple sclerosis or systemic lupus erythematosus (SLE) (7), there is a significant challenge for our healthcare systems.

CFS/ME manifests a wide range of symptoms, including fatigue, cognitive dysfunction, sleep disturbances, orthostatic intolerance, myalgia, and neuro-immuno-endocrine dysfunctions, which vary from patient to patient and can fluctuate over time. However, the hallmark of the disease is post-exertional malaise (PEM). PEM is a highly disabling and sometimes progressive symptom that occurs after physical or mental exertion or overload with a patient-specific threshold. The quality of life of those affected by ME/CFS can be severely limited: long courses and limited participation in social and professional life are not uncommon (8). A Danish study shows that ME/CFS patients have a lower quality of life than patients with cancer, stroke or multiple sclerosis (7) (9). The etiology of ME/CFS is not yet fully understood despite numerous efforts. It is conceivable that various molecular damage patterns lead to different clinical presentations, resulting in heterogeneity within the disease.

Several pathophysiological signatures in ME/CFS are discussed: e.g. energy metabolism and mitochondrial dysregulation (10, 11) and neuroendocrinological processes (12) have been implicated in ME/CFS. Furthermore, the involvement of gastrointestinal process, e.g. gut microbiota, enteric dysbiosis, and bacterial translocation has been suggested (13–15). Given this complexity, the classification as a syndrome is justified, and patient subtyping becomes a central task (16). Especially the gastrointestinal tract, skin, urogenital tract, and bronchopulmonary system, serve as habitats for microorganisms. These microorganisms, collectively referred to as the microbiota, represent a diverse community primarily composed of prokaryotic bacteria, archaea, microeukaryotes, and viruses. The microbiota interacts directly with the individual, governing physiological functions from maintaining local barrier homeostasis to regulating metabolism, hematopoiesis, immunity, and other systemic control circuits. In light of this, the term ‘holobiontic concept’ was recently introduced to better emphasize the significance of interactions between microbiota and humans (17). Studies by the American Human Microbiome Project and the European MetaHIT project demonstrate that the inter-individual variability, or beta diversity, of the gastrointestinal microbiota is considerably high in healthy individuals. Thus, there is no uniform ‘standard’ or ‘normal’ intestinal microbiota that can be defined. The stability of the gastrointestinal ecosystem appears to depend on the totality of microorganisms present, encompassing all required functions, with individual microorganisms being replaceable (18). Despite the substantial interindividual diversity of the microbiota, there is a high stability and convergence at the functional level (19). The intestinal microbial ecosystem exhibits functional redundancy, implying that various combinations of metabolically active bacterial species can fulfill the same function, allowing fundamentally different bacteria to perform similar or identical functions (20). In addition to bacteria and archaea, the microbiota contains approximately 10^9^ viruses per gram of stool. Sequencing studies in recent years have shown impressive inter-individual variability (beta diversity) with low diversity within the individual (alpha diversity). The enteric virome appears to be specific to each individual, with little or no convergence even in monozygotic twins or family members living in the same household. Most of the viral microbiota consists of previously uncharacterized viruses.

Microbiome research has evolved enormously during the past two decades and represents a new paradigm from which to approach many of the common diseases. It is well accepted, that the disturbances of the microbiota (dysbiosis) and its metabolome modulates host metabolism, inflammation, and immunity and plays a significant role in a number of gastrointestinal and extra-gastrointestinal diseases (21, 22). Although the changes underlying these diseases are becoming better understood, they remain far from sufficiently clarified. For various diseases such as chronic inflammatory bowel disease (IBD), *Clostridioides difficile* infections (CDI), gastrointestinal tumor diseases, liver diseases, and irritable bowel syndrome (IBS), it is evident that the gastrointestinal microbiota is characterized by reduced diversity (23, 24). The loss of this diversity is likely a potential risk factor for the development of these diseases.

There is growing evidence that the following gastrointestinal factors should be considered in ME/CFS:

- **Gut dysbiosis:** gut dysbiosis, which refers to an imbalance or disruption in the composition of the gut microbiota.
- **Gut-brain axis:** The gut-brain axis is a bidirectional communication system between the gut and the brain
- **Gut permeability and bacterial translocation:** Increased gut permeability, also known as “leaky gut,”.

In this mini review, we explore the proposed pathways between the gastrointestinal microbiome and ME/CFS. Overall, it is difficult to evaluate findings that were made in patients with “real” ME/CFS and that were found in patients with chronic fatigue, e.g. in the context of chronic IBD. In our review, we focus on findings of disruptions in the intestinal microbiome, with changes in the intestinal barrier leading to chronic inflammation with increased reactive oxygen species (ROS). We discuss the potential for therapeutic modulation of the microbiome in ME/CFS patients, although there is currently no convincing evidence for defined therapeutic concepts. While several questions remain unanswered, insights into microbiome modulation from studies in other diseases may have implications for modern therapeutic approaches to ME/CFS.

## Intestinal dysbiosis in ME/CFS

**Figure 1:**
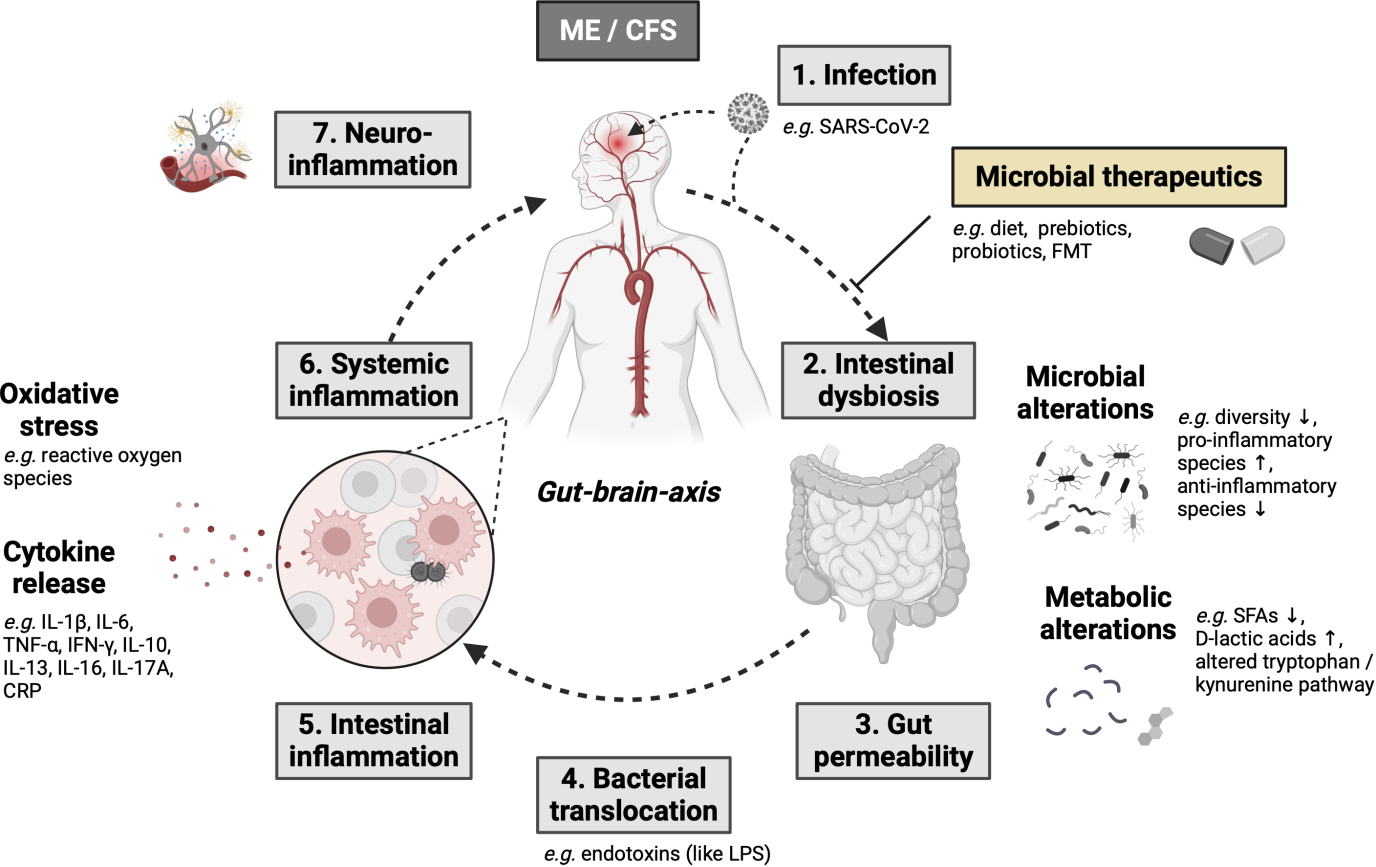
The gut-brain and microbiota-immune-axis as major molecular pathomechanisms resulting in ME/CFS development with potential treatment options based on the intestinal microbiome (modified after 26) (14, 33, 46, 47) Created with BioRender.com. Arrow up means "increase", arrow down means "decrease".

Changes in the intestinal microbiome that are associated with reduced diversity, a loss of intestinal commensal microorganisms and an increase in “pro-inflammatory” species are referred to as dysbiosis and significantly lead to an altered host response or immune modulation of the intestine in chronic fatigue syndrome patients (15). With the start of next-generation sequencing of stool samples, it was possible to prove that intestinal dysbiosis contributes to IBD (25–27) and could act as a driving force in the development of neurodegenerative diseases (such as Parkinson’s disease (28), Alzheimer’s disease (29), multiple sclerosis (30), amyotrophic lateral sclerosis (31) and Huntington’s disease (32). It is also suspected of triggering ME/CFS and long-COVID syndrome as a potentially reversible disease (33). Interestingly, a large proportion of ME/CFS patients (35 to 90%) have gastrointestinal complaints, which are often associated with the comorbidities IBS and IBD (34). Various studies have demonstrated intestinal dysbiosis in ME/CFS (**Table 1**) (13, 33, 34, 36–44), although uniform microbiome signatures are non-existent. This is mainly due to the high intra- and inter-individual variability of the microbiome. During homeostasis, the six phyla “Firmicutes, Bacteroidetes, Actinobacteria, Proteobacteria, Fusobacteria and Verrucomicrobia” (45) predominate; ME/CFS is often associated with a decrease in the Firmicutes phylum and an increase in the Bacteroidetes phylum. However, no specific microorganism has been identified aetiologically. Consequently, it is unclear to what extent the intestinal microbiome is pathogenetically responsible for the development of ME/CFS. Recent research point toward an involvement of the microbiota-immune-axis in ME/CFS (15). These microbiota-immune axis should be addressed by comparative studies characterized by long-term fatigue symptoms, including IBD, post-acute infection syndromes (e.g. post–COVID-19 condition). However, it is postulated that intestinal dysbiosis leads to immunometabolic alterations (*e.g.* reduced production of antimicrobial peptides, short-chain fatty acids (SCFAs), altered tryptophan/kynurenine pathway), which causes a disturbed intestinal barrier, increased bacterial translocation, consecutive systemic inflammation as well as neuroinflammation and neuroimmune dysfunction (**Figure 1**) (14, 46, 47). Further large cohort studies are lacking in order to better understand causal or functional relationships between the microbiome, neuroinflammation and neurocognitive diseases.

**Table 1:** Overview of microbial changes in patients with ME/CFS cases compared to healthy controls (HC) [modified after (33, 35)].

Study	Subjects	Year	Country	Intestinal dysbiosis	Reduced microbiota	Enhanced microbiota	Reference
1	2076 cases, 460,857 HC	2023	11 countries	Yes	None	Anaerobic bacteria (e.g. *Paraprevotella, Ruminococcaceae UCG_014)*	He et al., 2023 (36),
2	35 cases, 70 HC (Fukuda Criteria)	2021	Italy	Yes	*Firmicutes*	*Bacteroidetes*	Lupo et al., 2021 (13),
3	48 cases, 52 HC (Fukuda und International Consensus Criteria)	2020	Japan	Yes	None	*Blautia, Coprobacillus*, *Eggerthella*	Kitami et al., 2020 (37),
4	17 cases und 17 HC (Fukuda Criteria)	2018	USA	No	None	None	Mandarano et al., 2018 (38),
5	50 cases, 50 HC (Fukuda, Canadian Criteria)	2017	USA	Yes	*Faecalibacterium* (with IBS), *Bacteroides vulgatus* (without IBS)	*Alistipes* (with IBS), *Bacteroides* (without IBS)	Nagy-Szakal et al.2017 (34),
6	48 cases, 39 HC (Fukuda Criteria)	2016	USA	Yes	*Firmicutes* (n.s.), anti-inflammatory species (n.s.)	Pro-inflammatory species, *Proteaobacteria* (e.g. *Enterobacteriaceae)*	Giloteaux et al., 2016 (39),
7	1 case, 1 HC (34-year-old monozytogenic male twins) (Fukuda Criteria)	2016	USA	Yes	*Faecalibacterium, Bifidobacterium*	None	Giloteaux et al., 2016 (40),
8	34 cases and 25 HC (Canadian Criteria)	2016	Australia	Yes	Anaerobic bacteria, *Bacteroides* spp.	Aerobic bacteria (n.s.), *Clostridium* spp.	Armstrong et al., 2016 (41),
9	10 cases, 10 HC (Fukuda criteria)	2015	Italy	Yes	*Actinobacteria; Firmicutes* (n.s.)	*Bacteroidetes* (n.s.)	Shukla et al., 2015 (42),
10	35 cases, 36 HC (Fukuda criteria)	2013	Belgium	Yes	*Firmicutes*	*Bacteroidetes (e.g. Alistipes)*	Frémont et al., 2013 (43),
11	108 cases and 177 HC (Holmes, Fukuda and Canadian criteria)	2009	Australia	Yes	*E. coli*, Gram-positive/Gram-negative bacteria ratio	Aerobic bacteria, D-lactic acid producing: *E. faecalis*, *S. sanguinis*	Sheddy et al., 2009 (44),

IBS, irritable bowel syndrome, n.s., non significant.

## Intestinal barrier integrity in ME/CFS

The gastrointestinal barrier ensures the selective absorption of water, electrolytes, and nutrients while preventing the translocation of pathogenic organisms or their components from the intestinal lumen into the mucosa and its compartments. This barrier is a complex morphological-functional mechanism that involves the gut microbial barrier, mucus, gastrointestinal motility, secretion, epithelial barrier, and the immune system (both innate and adaptive). The intestinal microbiome significantly influences the integrity of this barrier in various ways. For instance, it can modulate immunological cascades or produce metabolites such as SCFAs. Disturbances in this delicate balance result in increased intestinal permeability (48). It is well known, that dysbiosis in the intestinal microbiome, triggered by antibiotic therapy, inflammatory diseases, or repeated or excessive alcohol consumption, can lead to a loss of integrity in the intestinal barrier, which is then referred to as “leaky gut”.

Undoubtedly, a leaky gut can trigger systemic chronic inflammatory reactions; inflammatory processes in the liver are a typical example (49, 50). This raises the question of whether a leaky gut is also important in ME/CFS. Various findings suggest this (42, 51–53). In a controlled study, Shukla and colleagues were able to detect a pathological bacterial translocation of 6 of 9 main bacterial genera after 72 hours after a defined stress test compared to 2 of 9 in controls (42). Moreover, there are increased specific IgA and IgA levels against LPS, a bacterial marker for gram-negative bacterial translocation, in patients with ME/CFS. This correlates with the severity of the disease (52, 53). Furthermore, there is evidence suggesting that the heightened translocation of LPS, leading to gut-derived inflammation, induces systemic inflammation and reactive nitrogen species (RNS) and reactive oxygen species (ROS) — a potential pathway in ME/CFS. Several studies report increased oxidative stress in ME/CFS (for review, see Missailidis et al.) (54).

## Microbiota-gut-brain axis in ME/CFS

The existence of a bi-directional communication and interaction between the gut and the brain is already known since the middle of the 19^th^ century. In recent years, there is growing evidence leading to the concept of a “gut-brain axis” (55) The communication of the gut and the microbiota is of great importance for several mechanisms, physiologic and pathophysiologic. For example, Sudo et al. were able to demonstrate the importance of the intestinal microbiome for the development of the hypothalamus-pituitary-adrenal axis and on stress response using germ-free animals (56). In another study, Bercik et al. have shown, that in two different strains of germ-free mice, fecal microbiota transplantation using feces of the own strain resulted in behavior similar to the own strain, while faecal microbiota transplant (FMT) using feces of the other strain resulted in a behavior similar to the other strain (57). Although the exact physiological pathways are still matter of debate in many cases, the relevance of the gut-brain communication in ME/CFS patients is supported by several studies. Noteworthy, until today the exact pathophysiologic mechanisms how the gut-brain-axis can influence neuropsychiatric symptoms remains unclear. Evidence of this interactions resulted from different studies (58–61) Basically, it can be linked via different ways (14). These pathways include changes in the immune system, including the cellular immune system, e.g., in regulatory T-cells (62), NK-cells (63), or CD8+ T-cells (64) as well as cytokine production, e.g., increased TGF-ß production (65) and immunoglobulins (52). Noteworthy, these results are still controversial as chronic inflammation may also be caused by an underlying disease (14, 47). Neurotransmitters play an important role in gut-brain interaction (66)and play a role in the development of psychological disorders like depression (67), In ME/CFS, levels of Tryptophan as a neurotransmitter influenced by the microbiome, were linked to ME/CFS (68) Finally, the gut microbiome was shown to have direct influence on nerval stimulation of the vagal nerve, which however may also be a bi-directional influence as the vagal nerve innervates the colon (reviewed at (69))

However, even if the direct modes of action are not known until today, there are several indirect hints underlining the importance of the gut-brain-axis in neuropsychiatric symptoms in general and ME/CFS in particular. A recent study reported a reduction in ME/CFS symptoms after rectal infusion of bacteria (70). Noteworthy, the majority of the patients (52/60) suffered from concomitant IBS as underlying disease. Although, therefore, it is obvious that the underlying IBS was treated, this study gives clear evidence for the importance of gut-brain communication in ME/CFS. Additionally, the modulation of the intestinal microbiome by antibiotic or probiotic treatment showed improvement in different neuropsychiatric symptoms as another indirect hint for the relevance of the gut-brain-axis (58–61, 71)

Metagenomic profiling revealed different clusters of faecal bacterial indicative for ME/CFS, with these clusters being different in patients with or without concomitant IBS (34) In patients with IBS, increased abundance of unclassified Alistipes and decreased Faecalibacterium were reported, while in patients without IBS, increased unclassified *Bacteroides* abundance and decreased *Bacteroides vulgatus* were biomarkers of ME/CFS. Additionally, the authors also found differences in metabolomic pathways, involving unsaturated fatty acid synthesis, atrazine degradation, vitamin B6 synthesis and pyrimidine ribonucleoside degradation. As the intestinal microbiome consists of bacterial and viral species, and give the fact, that ME/CFS is, despite a high number of unexplained cases, frequently reported as post-viral sequela such as after SARS-CoV-2 (72), another study focused on viral taxa in feces, blood and saliva, however, the authors did not observe any differences between ME/CFS patients and controls (73).

## Dysbiosis and the microbiota-gut-brain-axis

Dysbiosis, which refers to an imbalance or disruption in the composition of the gut microbiota, can impact the microbiota-gut-brain axis in ME/CFS (**Figure 1**). While the exact mechanisms are still being investigated, some potential ways how dysbiosis may influence the microbiota-gut-brain axis in ME/CFS are inflammation and immune activation, neurotransmitter signaling, metabolite generation, activation of the immune-brain axis.

- **Inflammation and immune activation:** Dysbiosis can lead to increased gut permeability, also known as “leaky gut,” allowing the translocation of bacteria or bacterial products from the gut into the bloodstream. This can trigger immune responses and systemic inflammation, which may affect the brain and contribute to the symptoms of ME/CFS (74).
- **Neurotransmitter signaling:** The gut microbiota has the ability to produce neurotransmitters and modulate their signaling. Dysbiosis can disrupt the production and balance of neurotransmitters, such as serotonin and gamma-aminobutyric acid (GABA), which play important roles in regulating mood, cognition, and other brain functions. Alterations in neurotransmitter production and signaling may contribute to the symptoms experienced by ME/CFS patients (75).
- **Metabolite generation**: The gut microbiota produces various metabolites such as SCFAs that can influence brain function and behavior. Dysbiosis can alter the production and availability of these metabolites, potentially affecting the gut-brain communication and contributing to the symptoms of ME/CFS.
- **Activation of the immune-brain axis:** Dysbiosis can activate the immune system, leading to the release of pro-inflammatory cytokines and other immune molecules. These immune molecules can communicate with the brain through various pathways, including the vagus nerve and immune cell trafficking, potentially influencing brain function and contributing to the symptoms of ME/CFS (76, 77).

## Modulation of the gastrointestinal microbiome as rational therapy in ME/CFS

In ME/CFS patients, the gut microbiome has less biodiversity compared to healthy individuals and this is thought to contribute to the onset and progression of ME/CFS (39). Therefore, microbiome modulation has received attention as a new therapeutic target. There are several potential therapeutic methods including dietary exclusions or prebiotics, probiotics, FMT and other modifications. However, when evaluating microbiota-modulating studies in ME/CFS, it is crucial to consider target parameters. Endpoints such as inflammation markers, cytokines, and lymphocyte subsets are of lesser importance compared to clinically relevant endpoints, such as an improvement in the quality of life.

Probiotics, live bacteria believed to promote health, have been recognized for their beneficial effects for many decades in different indications. The use of the probiotic *Escherichia coli (E. coli) Nissle* 1917 is recommended in guidelines for patients with ulcerative colitis (78). A pilot study by an Italian group demonstrated that the intake of various probiotics over 8 weeks led to a modification of well-being status, as well as inflammatory and oxidative indexes in CFS/ME patients, resulting in a reduction of inflammatory parameters after probiotic intake (13)(**Table 1**). However, the study’s limited evidence was due to a small sample size and an uncontrolled design. In a study focused on clinical endpoints, Sullivan et al. examined the effects of *Lactobacillus paracasei* ssp. *paracasei* F19, *Lactobacillus acidophilus* NCFB 1748, and *Bifidobacterium lactis* Bb12 on fatigue and physical activity in 15 CFS patients, who met the 1994 CDC criteria for CFS (60). After 4 weeks, neurocognitive functions improved during the study period, but there were no significant changes in fatigue and physical activity scores. At the end of the study, 6 out of 15 patients reported that they had experienced improvement. A systematic review summarizes the effects of probiotic treatment on gastrointestinal symptoms and typical IBS in patients with CFS/ME. The authors found that in 25 studies (including 24 randomized control trials) the evidence available for the use of probiotic interventions in CFS/ME was poor and limited (79). Further, the high variability in probiotic formulations makes it challenging to combine the results of clinical studies.

Overall, the evidence for the use of probiotics in ME/CFS is weak. Despite numerous working groups postulating a connection between the development and progression of ME/CFS and the microbiota, it is surprising that only two studies are currently listed on ClinicalTrials.gov examining the influence of probiotics on the disease (last query: 07-Oct-2023).

In recent years, FMT has been examined as a justified alternative to classic medication concepts for a variety of gastrointestinal and neurological diseases. There is no doubt that this concept is successful in CDI, ulcerative colitis or patients with IBS and is recommended as a proven therapy for CDI in international guidelines. In recent years, FMT has emerged also as a promising therapy for ME/CFS patients. The goal of this concept is to restore a healthy gut microbiota by introducing feces from a healthy donor into the recipient’s digestive system. Since ME/CFS is a systemic disease, it is not surprising that gastrointestinal symptoms are common (80). It is known that comorbidities such as IBS or Crohn’s disease occur more frequently in ME/CFS patients, which once again points to the causal importance of the intestinal microbiome in this clinical picture (81, 82). Against this background, the effects of taking prebiotics and probiotics were compared with FMT treatment over 10 days in a non-randomized study in patients with chronic fatigue syndrome. Each patient received 10 FMT, each from a different screened donor, and the transplant was delivered via a rectal catheter, into the lower part of the sigmoid colon. The effect was measured by patient self-assessment (no improvement, “0” to maximum improvement “100”). Of the 21 patients with FMT treatment, 17 reported an improvement of 65-95%, and most importantly seven patients reported a normalization of their quality of life and performance (83). However, a recently published small randomized, double blind, placebo-controlled pilot study demonstrated that FMT was safe but did not relieve symptoms or improve the health-related quality of life of patients with CFS. The small number of study subjects limits the generalizability of these results (84). With FMT, the aim is to transfer the donor microbiome as completely and as long-term as possible. An important consideration is that this study only conducted a single FMT (84). In ulcerative colitis (85), as well as in patients with IBS, effectiveness has been shown to increase with a higher number and duration of FMT cycles. This suggests that continuous application of the donor microbiome to achieve permanent remission is a subject of discussion. To address the optimal approach for engraftment, a metagenomic analysis of stool microbiomes from donors, pre-FMT recipients, and post-FMT recipients was performed (86). Different FMT methods were compared in a systematic meta-analysis involving 24 studies (86). This groundbreaking study found that the clinical response was correlated with the extent of engraftment. Furthermore, antibiotic treatment prior to FMT significantly improved engraftment. The type of FMT administration was most strongly associated with the success of FMT, particularly a combined type of FMT administration (a combination of upper and lower gastrointestinal applications and gastrointestinal administration). Bacteroidetes and Actinobacteria spp. showed higher average engraftment rates among strains compared to Firmicutes and Proteobacteria spp., while gram-positive bacteria were less likely to have high engraftment rates than more resilient gram-negative species. These findings impressively underscore the need for characterizing and, ideally, standardizing the FMT donor stool should be sought.

## Conclusion

In patients with ME/CFS, various studies have yielded inconclusive results regarding changes in the gastrointestinal processes where microbiota is involved. Whether these divergent findings are due to different molecular phenotypes of patients with ME/CFS remains speculative. Presently, dysbiosis in ME/CFS is to be understood as an association; causality is not proven from a critical perspective. Nevertheless, the growing comprehension of the interactions between the microbiome and the host presents an intriguing pathophysiological concept, forming the foundation for rational future therapeutic approaches. A randomized controlled study involving well-defined ME/CFS patients, encompassing post-exertional malaise (PEM), and employing repeated and long-term faecal microbiota transplantation (FMT), appears to be the most promising approach to establish causality. Such understanding of interactions will lead to concepts that help overcome therapeutic nihilism.

## Author contributions

AS: Conceptualization, Writing – original draft, Writing – review & editing. SQ: Writing – original draft, Writing – review & editing. CP: Writing – original draft, Writing – review & editing. PR: Writing – original draft, Writing – review & editing.
